# Reliability and Educational Value of YouTube Videos of Complete Meso-Colic Excision With Right Hemicolectomy in the COVID-19 Pandemic

**DOI:** 10.7759/cureus.25387

**Published:** 2022-05-27

**Authors:** Mustafa Y Uzunoglu, Omer Yalkin

**Affiliations:** 1 General Surgery, Bursa City Hospital, Bursa, TUR; 2 Surgical Oncology, Bursa City Hospital, Bursa, TUR

**Keywords:** video analysis, covid-19, youtube, right hemicolectomy, complete mesocolic excision

## Abstract

Aim: To investigate the reliability and educational value of YouTube videos of minimally invasive complete mesocolic excision with right hemicolectomy procedures.

Materials and methods: We searched YouTube with the terms “Laparoscopic and Robotic Complete Mesocolic Excision with Right Hemicolectomy” on January 12, 2021. To assess the reliability of the videos, we evaluated nine steps in each video and scored the videos based on the key steps they contained. The videos were divided into three groups according to the source of the upload. The total number of views, length, time since upload, and the number of likes, dislikes, and comments were recorded for each video. Narration, the use of descriptive subtitles, and the upload status by an expert surgeon were also examined.

Results: Sixty-eight videos were included in the study. A positive significant correlation was identified between the comprehensiveness score (CS) and the number of views (p=0.025). The CSs of the videos accessed from academic channels, as well as those accessed from journals, congress, and association channels, recorded higher CSs than those obtained from the personal channels of consultants (p=0.003). It was also found that CSs were higher in the videos of expert surgeons (p<0.001) and narrated videos (p<0.001).

Conclusion: Not all YouTube videos on this subject have reliability and educational value. Surgical videos on YouTube may be evaluated by a video review commission formed by academic institutions, surgical associations, or expert surgeons, and videos suitable for education could be brought together and published via a free channel.

## Introduction

Colorectal cancer is the third most common type of cancer in both men and women. According to Global Cancer Observatory (GLOBOCAN) data, there are approximately two million new cases per year worldwide [1]. The effect of surgical operations on survival has been emphasized in many studies, with cancer-related mortality reported to be reduced significantly due to advances in surgical techniques over the years. Colon cancer patients who undergo complete mesocolic excision and effective lymph node dissection have a higher long-term survival rate [2]. 

The COVID-19 pandemic has had a severe impact on the quantity and quality of surgical training. A study conducted by Purdy et al. reported that there had been a 33.5% reduction in the total number of major operations performed by 1,358 general surgery residents during the pandemic period when compared to the pre-pandemic period [3]. With the interruptions to face-to-face education, live or video-recorded presentations have come to replace bedside applications and training meetings. Furthermore, elective surgeries have been suspended along with any procedures other than emergency surgical operations. It is considered that monitoring the operations of different expert surgeons at different centers may complement the traditional approach to surgical education [4]. YouTube, as the most widely used video-sharing site, is used as a source of surgical videos by many surgical assistants, medical students, and even surgeons. The present study investigates the reliability and educational value of the minimally invasive complete mesocolic excision with right hemicolectomy videos uploaded to YouTube.

## Materials and methods

A search on YouTube was conducted using the search terms “Laparoscopic Complete Mesocolic Excision with Right Hemicolectomy” and “Robotic Complete Mesocolic Excision with Right Hemicolectomy’’ on January 12, 2021, and the search results were sorted using the filter “Sort by View Count.” It was assumed that filters such as “medical student” and “surgical trainee,” and videos with a high number of views and interactions, would be of higher quality for medical education and the visualization of surgical techniques. The videos were saved in a playlist as the search results on YouTube can change from day to day, after which duplicate videos (n=6) and videos without audio narration in English (n=3) were removed. Videos in multiple parts were treated as a single video (n=1). As a result of the search, 68 videos were obtained, 46 of which were of laparoscopic procedures, and 24 were of robotic complete mesocolic excisions. To assess the reliability of the videos, we evaluated nine steps in each video, based on the steps detailed in an article published by Strey et al. with some modifications [5]. The videos were watched together with three surgeons with experience in oncological surgery, and each video was scored according to whether or not it contained the nine key steps (0 - contains none at all, 1 - contains some, 2 - contains all). The videos were divided into three groups based on their comprehensiveness scores: poor for <9, moderate for 10 to 13, and good for >14. Each surgeon evaluated the videos separately and in case of disagreement, the evaluation was made with the consensus of the three surgeons (Table 1).

**Table 1:** Distribution of answers to the questions SMV: Superior mesenteric vein; SMA: Superior mesenteric artery;  ICA: Ileocolic artery; ICV: Ileocolic vein; MCA: Middle colic artery; MCV: Middle colic vein; RCA: Right colic artery; RCV: Right colic vein; RGEV: Right gastroepiploic vein; SRCV: Superior right colic vein; GEV: Gastroepiploic vein; ASGDV: Anterior superior gastroduodenal vein

1- Is patient positioning and trocar localization described?
2- Are SMV, SMA, and ICA identification described?
3- Are Henle Trunk, SRCV, RGEV, RCV, RCA, MCA, MCV, ASGDV, and GEV identification described?
4- Is the ligation of the ICA, ICV, RCV, RCA, SRCV, and right branch of the MCA described?
5- Is the preservation of the visceral fascia without injuring the mesocolon described?
6- Is the division of the gastrocolic ligament and the Lesser sac entry described?
7- Is the division of the lateral attachments of the right colon described?
8- Is anastomose described?
9- Is the appearance of the specimen described?

The videos were divided into three groups based on the source of the upload. Source 1: Academia-based channels (university and training and research hospitals). Source 2: Educational videos uploaded by specialized (colorectal) surgical associations, colorectal surgery congress videos, and YouTube video channels of Colorectal Journals. Source 3: Consultant videos (uploaded by an individual consultant with no affiliation).

The total number of views, length, and time since upload, as well as the numbers of likes, dislikes, and comments, were recorded for each video. Viewers' interactions with the videos were calculated in terms of “views per day” (calculated as the total number of views divided by the number of days since uploaded).

The videos were also examined for narration, descriptive subtitle presence, and upload status by an expert surgeon. The criterion according to which the surgeons were classified as experts was having at least five publications on minimally invasive colorectal surgery listed in PubMed within the last two years.

Statistical method

The consistency of coverage score to normal distribution was examined with the Shapiro-Wilk test. The scope score was reported as a symptom statistically with the median, interquartile range, minimum and maximum values. In comparing the coverage score, Mann-Whitney U and Kruskal Wallis tests were used, and the subgroup analyses were made using the Dunn Bonferroni test after the Kruskal Wallis test. The relationships between the scope score and other discrete and continuous variables in the study were examined by correlation analysis, and the Spearman correlation coefficient was calculated. Statistical analyses were performed using Statistical Package for Social Sciences (SPSS) version 23 (IBM Corp., Armonk, NY, USA). A type I error rate of 5% was considered statistically significant for all statistical comparisons.

## Results

Of the 68 videos watched, 46 were laparoscopic and 23 were robotic complete mesocolic excision videos. The distribution of responses to the items used in the comprehensiveness calculation is presented in Table 2.

**Table 2:** Distribution of responses to the items Data were presented as n (%), SMV: Superior mesenteric vein; SMA: Superior mesenteric artery;  ICA: Ileocolic artery; ICV: Ileocolic vein; MCA: Middle colic artery; MCV: Middle colic vein; RCA: Right colic artery; RCV: Right colic vein; RGEV: Right gastroepiploic vein; SRCV: Superior right colic vein; GEV: Gastroepiploic vein; ASGDV: Anterior superior gastroduodenal vein

Item	Not Mentioned (0)	Briefly mentioned (1)	Mentioned in detail (2)
Patient position and port placement	30 (44.10%)	8 (11.80)	30 (44.10%)
SMV, SMA, ICA identification	1 (1.50%)	13 (19.10%)	54 (79.40%)
Henle Trunk, SRCV, RGEV, RCV, RCA, MCA, MCV, ASGDV, GEV identification	16 (23.50%)	16 (23.50%)	36 (53%)
Ligation of ICA, ICV, RCV, RCA, SRCV, right branch of MCA,	4 (5.90%)	12 (17.60%)	52 (76.50%)
Preservation of visceral fascia without injuring mesocolon	0	13 (19.10%)	55 (80.90%)
Division of gastrocolic ligament and Lesser sac entry	15 (22.10%)	12 (17.60%)	41 (60.30%)
Division of lateral attachments of colon	3 (4.40%)	8 (11.80)	57 (83.80%)
Anastomosis	19 (27.90%)	12 (17.60%)	37 (54.40%)
View of specimen	36 (52.90%)	12 (17.60%)	20 (29.40%)

A comparison of the comprehensiveness scores obtained for each of the 68 videos based on the source of the video, the expertise of the surgeon in the video, the narration in the video, and the presence of a descriptive subtitle in the video is presented in Table 3. When Table 3 is examined, the comprehensiveness score can be seen to differ depending on the upload source (p=0.001).

**Table 3:** Comparison of comprehensiveness scores Data presented as median (interquartile range). a: Kruskal Wallis test, b: Mann-Whitney U Test

Source	Comprehensiveness Score
Academia-based university and educational and research hospital channels (n=4)	17.50 (1.75)
Journal, Congress, Association Channels (n=28)	14 (16)
Personal channels of consultants (n=36)	10 (5.50)
p-value	0.001^a^
Expert Surgeon	
Yes (n=40)	14 (2)
No (n=28)	10 (3)
p-value	<0.001^b^
Narration	
Yes (n=38)	15 (4.50)
No (n=30)	10 (5)
p-value	<0.001^b^
Descriptive Subtitle	
Yes (n=42)	14 (6)
No (n=26)	11.50 (6.25)
p-value	0.210^b^

It was determined that the comprehensiveness scores differed based on whether the surgeon in the video was an expert or not (p<0.001). The comprehensiveness scores of the videos featuring an expert surgeon (14( [minimum-maximum: 7 to 18]) were higher than those of videos that did not feature an expert surgeon (10 [min-max: 2 to 18]) (p<0.001).

Similarly, the comprehensiveness scores were found to be higher for narrated videos (15 [min-max: 7 to 18]) than for non-narrated videos (10 [min-max: 2 to 16]) (p<0.001).

It was noted, however, that the comprehensiveness score was unaffected by the presence or absence of descriptive subtitles in the video (p=0.210). The median comprehensiveness score of videos supported by descriptive subtitles was 14 (min-max: 7 to 18) and 11.50 (min-max: 2 to 18) for those not supported by subtitles.

Table 4 presents the relationship between comprehensiveness score and the number of views, length, time since uploading and the total number of views, and the effect on the comprehensiveness score of the number of likes and comments on the videos.

**Table 4:** Comparison of comprehensiveness scores Data presented as median (interquartile range). a: Kruskal Wallis test, b: Mann-Whitney U Test

Number of views	Comprehensiveness Score
r_s_	0.27
p-value	0.025
Video Length (min)	
r_s_	0.07
p-value	0.954
Time since uploaded (days)	
r_s_	-0.03
p-value	0.826
Total Views	
r_s_	0.26
p-value	0.035
Dislike	
Yes (n=23)	14 (4)
No (n=45)	12 (6)
p-value	0.202^b^
Comment	
Yes (n=16)	14 (5)
No (n=52)	14 (6)
p-value	0.844^b^

The median number of views of the videos included in the study was 638.50 (min-max: 10 to 12,225), and a significant positive correlation was found between the comprehensiveness score and the number of views (rs=0.27, p=0.025). The comprehensiveness score of the videos can be expected to increase with an increase in the number of views. The median video length value of the videos included in the study was 9.08 minutes (min-max: 3.43 to 75.08), and there was no correlation between the comprehensiveness score and video duration (p=0.954). The time since the upload date of the videos included in the study was calculated as 933 (min-max: 13 to 2310) days. It was determined that the upload date of the video did not affect the comprehensiveness score (p=0.826). The median number of views of the videos was determined as 0.84 (min-max: 0.02 to 28.74).

It was observed that the comprehensiveness score was unaffected by the number of dislikes of the videos (p=0.202). The median comprehensiveness score of the videos with dislikes was 14 (min-max: 9 to 18) and the comprehensiveness score of the videos with no dislikes was 12 (min-max: 2 to 18).

Similarly, the comprehensiveness score was unaffected by video comments (p=0.844). The median comprehensiveness score of the videos with comments was 14 (min-max: 9 to 18) and the comprehensiveness score of the videos with no comments was 14 (min-max: 2 to 18).

## Discussion

YouTube is a giant Internet video broadcasting platform that attracts more than one billion video views per day and more than 500 hours of video uploaded per minute [6]. Some of the videos uploaded to YouTube have medical content. Although many sites on the Internet contain medical video archives, 95% of surgical residents prefer YouTube for their searches of surgical case presentations and technical videos [7]. Heterogeneous upload sources utilizing the Web 2.0 technology of YouTube prevent the standardization of video quality. Numerous studies have been conducted on YouTube content [8-11]. Similar to the findings of other studies, it was found in the present study that the likes, dislikes, number of comments, upload date, and video length did not affect the comprehensiveness score. In addition, it was determined that association, journal, and congress-sourced videos recorded high comprehensiveness scores and that the comprehensiveness score was higher for videos with narration and featuring an expert surgeon [12]. In the present study, a positive significant correlation was identified between the comprehensiveness score and the number of views. The reason for the high comprehensiveness score of videos sourced from academia or associations, congresses, and journals was concluded to be related to the greater number of interactions than the videos of consultants, and it was thought that viewers would consider the videos of other groups to be more reliable than those of unnamed surgeons. 

The working hours of surgical residents are limited to 80 hours a week in the United States and 48 hours a week in Europe, and consequently, those in Europe perform fewer surgeries during surgical training [13]. Residents have to work in the pandemic clinics rather than their clinics due to the shortage of consultants and surgical teams due to the COVID-19 pandemic. The negative impact on global healthcare systems of the closure of academic institutions due to the pandemic should not be underestimated [14]. Considering all the above, the COVID-19 pandemic can be seen to have had an enormous impact on the quantity and quality of training, for both medical and surgical residents, and this problem can be more clearly understood from the difficulties encountered when medical students and residents graduate without adequate training and must serve at the forefront of the field [15].

In operations that require a more specific and more effective learning process, such as oncological surgery, there has been a growing preference for learning techniques that can reduce the disadvantages associated with the pandemic. These include virtual online lectures and didactics, video and teleconferencing, interactive online surgical platforms and VR-simulators, live interactive virtual rounds, laparoscopic box training, homemade surgical simulation models, and video-based online platforms (YouTube, Websurg, Surgery Squad, etc.) [16].

It is thought that e-learning will make important contributions to the field by compensating for the deficiencies in theoretical and practical training. Jayakumar et al. listed the advantages of e-learning as easy access, learning flexibility, easy updating, multimedia presentation, and personalized learning, while the disadvantages were stated as dependence on internet speed, high initial cost, and the need for programming expertise [13]. Surgical education has begun to change from the Halstedian model with the innovations and advantages of the age [17]. We believe that instead of observing or performing an operation using the same technique on standard anatomy many times during training, it will be more beneficial for learning to see anatomical variations and challenging cases, and the approaches of different surgeons with different operating techniques, and to participate in these operations.

In the study conducted by Yee et al., it was stated that the factors affecting the quality of a video presentation can be divided into three groups. The first group includes factors related to the surgeon (level of experience, surgical specificity, familiarity with the operation, preparation process), the second group includes factors related to the procedure (duration of the case, complexity, frequency of operation), and the third group includes factors related to the purpose of the video (learning a surgical technique, teaching a surgical technique, publication in journals and textbooks, conference presentations) [18]. We believe that preparing videos according to these criteria will maximize the quality of the video.

Study limitations

This study has several limitations. Only English videos were included. The videos were filtered according to view count. As is known, video count is a dynamic process and can change every moment. The videos included only laparoscopic or robotic surgeries. Watching the videos together might have caused some bias, but this was eliminated with the consensus of the three surgeons.

## Conclusions

The results of this study indicate that the reliability and quality of health-related videos on YouTube are insufficient. In the future, surgical videos on YouTube may be evaluated by a video review commission formed by academic institutions, surgical associations, or expert surgeons, and videos suitable for education could be brought together and published via a free channel. Furthermore, financial support may be provided to encourage content creators to make more useful videos. In addition, new policies and strategies should be developed to audit health-related content uploaded to YouTube before it can be published.
